# Psychosocial predictors of early postpartum depressive and anxious symptoms in primiparous women and their partners

**DOI:** 10.1186/s12884-023-05506-8

**Published:** 2023-03-27

**Authors:** Erin J. Henshaw, Marie Cooper, Teresa Wood, Stacey N. Doan, Sanchita Krishna, Marie Lockhart

**Affiliations:** 1 Department of Psychology, Denison University, 100 W. College St, Granville, OH 43023 USA; 2 Riverside Methodist Hospital, OhioHealth, 3535 Olentangy River Rd., Columbus, OH 43214 USA; 3 Nursing Operations, OhioHealth, Columbus, USA; 4 Claremont McKenna College, 888 N. Columbia Ave, Claremont, CA 91711 USA; 5 OhioHealth Research Institute, 3535 Olentangy River Rd, Columbus, OH 43214 USA

**Keywords:** Postpartum depression, Fathers, Couples, Anxiety, Relationship satisfaction, Parenthood, Stress, Paternal depression, Couples, Parents

## Abstract

**Background:**

While the majority of research on postpartum depressive and anxious symptoms has focused on mothers, a growing body of research suggests a need to understand the role of the partner’s health and relationship quality as predictors of postpartum maternal depression, while also better understanding correlates of partner or paternal depression in the postpartum period. The purpose of the current study is to evaluate mother and partner stress, anxiety, mood, infant care support, and relationship quality as predictors of perinatal depressive and anxious symptoms in first time mothers and partners during the postpartum hospital stay.

**Methods:**

First time parent couples (n = 116) completed a survey during the two-day postpartum stay in a Midwest hospital. Depressive (EPDS) and anxiety symptoms (DASS-21-Anxiety) were assessed in both mothers and partners. Hierarchical linear regression was used to evaluate relationship satisfaction, partner infant care support, stress, and co-parent mood as predictors of mood in mothers and partners separately.

**Results:**

Stress was a predictor of anxiety and depression symptoms in both mothers and partners. Additionally, co-parent anxiety significantly predicted anxiety in both mothers and partners. Maternal relationship satisfaction was a predictor of the partner’s depressive symptoms, and maternal perceptions of partner infant support predicted maternal depressive symptoms.

**Conclusions:**

Together, these results suggest that stress, relationship satisfaction, and co-parent mood are related to depressive and anxious symptoms in mothers and partner, underscoring the need to continue exploring mother and partner mental health in a dyadic framework.

## Background

Perinatal depression and anxiety are common and problematic complications of childbirth that are associated with poorer health outcomes for infants and women. Perinatal depression (major and minor) and anxiety disorders are estimated to affect 11–13% of women [1, 2]. A recent meta-analysis of maternal-infant dyads reports that depression and anxiety are associated with poorer infant cognitive, socio-emotional, and motor development [3]. Additional associated risks to infants and women include poor bonding, low birth weight, less exclusive breastfeeding, and infant and maternal sleep problems [4].

Several risk and protective factors have been identified for perinatal depression and anxiety in women. A systematic review [5] identified maternal life stress, low social support, and domestic violence to be significant risk factors associated with perinatal depression. These factors were again identified in a review in 2016 [6] as predictors of both antenatal anxiety and depression, along with low partner support, mental illness history, and pregnancy complications. While postpartum anxiety has been studied less often, limited longitudinal evidence suggests a predictive role for marital satisfaction in postpartum maternal anxiety symptoms [7].

Though previously understudied, recent research has explored the prevalence and risks of depression and anxiety symptoms in fathers as well. A recent meta-analysis of 47 studies found a prevalence in fathers of 9.7% prenatally and 8.75% postpartum [8]. However, little is known about predictors of depression and anxiety in men during the perinatal period, but some risk factors have emerged. These include past depression history, parenting, pregnancy, or birth-related anxiety or stress, lower age, income, and education [9–13].

Among parents who are partnered, the relationship quality and the mental health of the partner have also emerged as potential risk factors for both maternal and paternal perinatal depression. For example, in longitudinal studies, paternal depression has been associated with paternally-reported relationship quality and satisfaction [10–12, 14]. A Delphi consensus study on paternal depression found key themes of risk factors including mother’s mood and marital conflict [15]. Partner’s depression score significantly predicted paternal depression [9].

Although most research has focused on maternal or paternal self-report only, a small number of studies have looked at maternal and paternal dyads to determine the role of partner symptoms and relationship quality. In a recent study of couples in the early postpartum period, parental stress, partner’s depressive symptoms, and birth-related distress predicted depressive symptoms in mothers and fathers. Notably, relation satisfaction predicted fathers’ depressive symptoms, but not mothers’ [9]. In contrast, two other recent studies of couples found that insufficient relationship support increased risks of postpartum depression in both mother and partner [16, 17].

The purpose of the current study is to evaluate stress, partner anxiety, mood, and relationship quality as predictors of perinatal depressive and anxious symptoms in first time mothers and partners during the postpartum hospital stay. It is expected that depressive and anxious symptoms in both mothers and partners will be positively predicted by stress and co-parent depressive and anxious symptoms and will be inversely predicted by relationship quality factors.

## Methods

### Procedure

Mother-partner pairs were recruited in person during the postpartum stay in a Midwest US hospital, between 1 and 2 days post-delivery. University and hospital IRB approval was obtained for all study activity (IRB # 1296284-21). All participants provided written informed consent, and surveys were completed separately by mother and partner on electronic tablets during the hospital stay. Current study survey data was collected as a pre-randomization baseline assessment for an ongoing clinical trial of a breastfeeding intervention (clinicaltrials.gov #NCT04578925), and inclusion/exclusion criteria were determined by the trial design. Enrollment occurred between February 2020 and February 2022, with multiple extended breaks in enrollment due to Covid-19 safety protocols at the hospital.

### Participants

Participants were eligible if they were age 18 or older, English speaking, primiparous, exclusively breastfeeding, and living together. Exclusion criteria included maternal current major depressive episode, as determined by the Maternal Mood Screener [18], or infant in Neonatal Intensive Care Unit. A total of 116 mother-partner pairs completed the survey.

### Measures

*Edinburgh Postnatal Depression Scale* [19]. The EPDS is a well-validated 10-item self-report depression screening tool, in which endorsement of each item is based on how participants feel during the previous 7 days. Possible scores range from 0 to 30, with high scores reflecting more depressive symptoms. The EPDS has shown high sensitivity, specificity, and positive predictive power for postpartum depression when using the 10 + score cutoff in US samples [19–22]. The EPDS has also been validated as an acceptable screening tool for men in the postpartum period [23, 24].

*Maternal Mood Screener* [18]. The mood screener questions were adapted from the Diagnostic Interview Schedule [25] and assesses lifetime and current major depressive episode. Positive endorsement of 5 out of 9 depression symptoms present for at least two weeks screens positive for possible major depressive episode. The screener is a self-report checklist using DSM-IV criteria that has shown high (kappa = 0.76) concordance with the SCID-CV for depression [26].

*DASS Anxiety scale* (DASS-21-Anxiety). The DASS-21 was used to measure anxiety. The anxiety subscale has shown high convergent validity with the Beck Anxiety Inventory [27].

*Postpartum Partner Support Scale* [20]. The PPSS is a 24-item self-report instrument to assess maternal perceptions of partner postpartum-specific support around infant care. Items are rated on a 4-point Likert-type scale (responses from 1 to 4) to produce a total score ranging from 24 to 96, with higher scores indicating higher levels of postpartum partner support. This scale demonstrated reliability with mothers during the postpartum period (Cronbach alpha = 0.96) and significantly distinguished between women with and without depressive symptoms at 8 weeks postpartum [20].

*Social Provisions Checklist* [28]. This is a 30-item measure of perceived relationship support from the co-parent, specifically. The scale covers six provisions of support: guidance, reliable alliance, reassurance of worth, attachment, social integration, and opportunity for nurturance. All items are rated on a 5-point scale for a total score of between 30 and 120, with higher scores indicating higher levels of support. This measure has been used in the early postpartum period (4 weeks) showing good reliability (Cronbach’s alpha = 0.82) and predictive validity, successfully distinguishing between women with and without depressive symptoms at 8 weeks postpartum [20].

### Analysis

T-tests (for continuous) and Chi Square (for nominal) analyses were used to examine differences in depression and anxiety scores on demographic variables. Correlations were used to examine relationships among all model variables. Hierarchical multiple regression was conducted to examine the relationships between hypothesized variables and depression and anxiety scores.

## Results

A total of 116 mother-partner pairs completed the survey. Participant characteristics are summarized in Table 1. No missing data was found within the 116 participant pairs. The majority of couples were married (92.2%), white (84.5% mothers, 81% partners), and heterosexual (96.6% mothers, 95.7% partners).

Demographic factors were analyzed for differences in all outcome variables. Results of t-tests suggest no significant differences in anxiety or depression symptoms by relationship status, delivery type, maternal or partner age, maternal race, partner race, and education level. Gender and sexual orientation could not be compared due to n < 5 in comparison categories.

**Table 1:** Participant Characteristics

		Mother (n = 116)		Partner (n = 116)	
		n (*M*)	% (SD)	n (*M*)	% (SD)
age		(30.04)	(4.05)	(31.1)	(4.69)
race/ethnicity					
	Asian/Asian-American	11	9.5	7	6
	Black/African-American	3	2.6	9	7.8
	Latinx	7	6	7	6
	White	98	84.5	94	81
	American Indian	0	0	0	
	Pacific Islander	0	0	0	
	Other	0	0	1	0.9
	Prefer not to respond	0	0	1	0.9
gender					
	Female	114	98.3	2	1.7
	Male	1	0.9	114	98.3
	Prefer not to identify	1	0.9	0	0
sexual orientation					
	Heterosexual or straight	112	96.6	111	95.7
	Gay or lesbian	1	0.9	1	0.9
	Bisexual	2	0.9	2	1.7
	Prefer not to answer	1	0.9	2	1.7
education level					
	Some high school	0	0	3	2.6
	High school diploma or GED	14	12.1	11	9.5
	Associate/technical degree	3	2.6	9	7.8
	Bachelor’s degree	59	50.9	58	50
	Graduate/professional degree	39	33.6	35	30.2
	Prefer not to answer	1	0.9	0	
relationship status					
	Living together not married	9	7.8		
	Married	107	92.2		
delivery					
	Vaginal	78	67.2		
	C-section	34	29.3		
	Assisted (forceps, vacuum)	4	3.4		
household income					
	<$24,999	3	2.6		
	$25,000–49,999	5	4.3		
	$50,000–74,999	14	12.1		
	$75,000–99,999	17	14.7		
	$100,000-124,999	23	19.8		
	Prefer not to answer	46	39.7		

Significant negative relationships were found between income and both maternal anxiety (r = − .28, p = .003) and partner anxiety (r = − .21, p = .027), suggesting that lower income was associated with more anxiety in both mothers and their partners. Based on these findings, income was included in all regression models as a step 1 variable.

Descriptive statistics and bivariate correlations for all mood and relationship variables are found in Table 2. Significant moderate relationships among depression, anxiety, and stress measures were found for mothers and for partners. Additionally, relationship satisfaction was significantly associated with depression, anxiety (in partners only), and stress.

**Table 2:** Descriptive Statistics and Bivariate Correlations (n = 116)

	M (SD)	1.	2.	3.	4.	5.	6.	7.	8.
1. maternal depression	7.17 (3.45)	1							
2. partner depression	6.14 (3.72)	0.13	1						
3. maternal anxiety	2.66 (2.59)	0.48**	0.08	1					
4. partner anxiety	1.79 (2.30)	0.16	0.49**	0.23*	1				
5. maternal stress	5.46 (3.5)	0.47**	0.10	0.60**	0.14	1			
6. partner stress	4.36 (2.78)	0.10	0.56**	0.05	0.65**	0.11	1		
7. maternal relationship satisfaction	4.69 (0.36)	− 0.25**	− 0.37**	− 0.10	− 0.28**	− 0.22*	− 0.32**	1	
8. partner relationship satisfaction	4.56 (0.49)	− 0.10	− 0.27**	− 0.02	− 0.29**	− 0.14	− 0.27**	0.36**	1
9. Postpartum partner infant support	3.74 (0.37)	− 0.31**	− 0.11	− 0.02	− 0.13	− 0.04	− 0.24**	0.50**	0.14

Note: Maternal and partner depression = Edinburgh Postnatal Depression Scale; Maternal and Partner anxiety = DASS-21 Anxiety scale. Maternal and partner relationship satisfaction = Social Provisions Checklist. Partner infant support (reported by mothers only) = with Postpartum Partner Support Scale. p < .05*, p < .01**

*Maternal anxiety.* Hierarchical multiple regression was used to estimate the contribution of maternal, partner, and relationship factors in predicting maternal anxiety (as measured by the DASS-21 Anxiety scale). In order to evaluate the unique contributions of partner and relationship factors, a 2-step hierarchical multiple regression was used, entering previously established predictors in the first step (income, maternal depressive symptoms and history), followed by a second step including maternal stress, perceived infant support from partner, and relationship satisfaction, as well as partner stress, mood, and relationship satisfaction. Residual scatterplots were visually inspected to determine that necessary assumptions were met.

Addition of the stress, relationship, and partner variables significantly improved the model (r^2^*Δ* = 0.22, p < .001). In the final model, three significant predictors of maternal anxiety were maternal depression β = 0.25, p = .007), maternal stress (β = 0.48, p < .0001), and partner anxiety (β = 0.22, p = .03; Table 3).

**Table 3:** Linear Regression Predicting Maternal and Partner Anxiety

Maternal Anxiety	B	SE B	β	R^2^_adj_
model 1				0.23
income	− 0.04	0.02	− 0.14	
maternal depression	0.05	0.01	0.44***	
maternal depression history	0.00	0.10	0.00	
model 2				0.42***
income	− 0.02	0.02	− 0.09	
maternal depression	0.03	0.01	0.25***	
maternal depression history	0.03	0.09	0.02	
postpartum partner infant support	0.07	0.09	0.07	
maternal relationship satisfaction	0.07	0.10	0.07	
maternal stress	0.35	0.06	0.48***	
Partner relationship satisfaction	0.07	0.06	0.09	
partner stress	− 0.07	0.07	− 0.11	
partner depression	0.00	0.01	0.00	
partner anxiety	0.24	0.11	0.22*	
Partner Anxiety	B	SE B	β	R^2^_adj_
model 1				0.29
income	− 0.05	0.02	− 0.20*	
partner depression	0.04	0.01	0.45***	
partner depression history	0.12	0.08	0.13	
model 2				0.54***
income	− 0.05	0.02	− 0.20*	
partner depression	0.01	0.01	0.14	
partner depression history	0.08	0.07	0.09	
postpartum partner infant support	0.10	0.08	0.11	
maternal relationship satisfaction	− 0.02	0.09	− 0.02	
partner relationship satisfaction	− 0.05	0.05	− 0.07	
partner stress	0.33	0.05	0.56***	
maternal stress	− 0.08	0.06	− 0.12	
maternal depression	0.00	0.01	0.01	
maternal anxiety	0.19	0.08	0.21*	

*Partner anxiety.* Similar methods were utilized to test a regression model of partner anxiety. In step 1, income and depressive symptoms were significant predictors. Step 2 significantly improved the model (r^2^*Δ* = 0.23, p < .001). Significant predictors in the second step included partner stress (β = 0.56) and maternal anxiety (β = 0.21). See Table 3.

*Maternal depressive symptoms.* Regression models were evaluated with maternal depression using the EPDS as a continuous outcome (Table 4). Maternal anxiety and depression history were significant predictors in step 1. The model was significantly improved by the addition of step 2 variables (r^2^*Δ* = 0.11, p = .009). Significant predictors included maternal stress (β = 0.26, p = .009) and perceived infant support, β = − 0.30, p = .001).

**Table 4:** Linear Regression Predicting Maternal and Partner Depressive Symptoms

Maternal Depression	B	SE B	β	R^2^_adj_
model 1				0.28
income	− 0.33	0.20	-0.13	
maternal anxiety	3.91	0.78	0.41***	
maternal depression history	2.46	0.84	0.24**	
model 2				0.40***
income	− 0.09	0.21	-0.04	
maternal anxiety	2.59	0.94	0.27**	
maternal depression history	2.22	0.82	0.21**	
postpartum partner infant support	-2.84	0.87	-0.30**	
maternal relationship satisfaction	0.52	1.03	0.05	
maternal stress	0.03	0.60	0.01	
Partner relationship satisfaction	1.84	0.69	0.26**	
partner stress	− 0.27	0.70	-0.04	
partner depression	0.06	0.09	0.06	
partner anxiety	0.35	1.14	0.03	
Partner Depression	B	SE B	β	R^2^_adj_
model 1				0.29
income	0.34	0.21	0.13	
partner anxiety	4.92	0.92	0.45***	
partner depression history	2.37	0.84	0.24**	
model 2				0.38**
income	0.28	0.22	0.11	
partner anxiety	1.86	1.18	0.17	
partner depression history	1.84	0.82	0.18*	
postpartum partner infant support	1.14	0.94	0.11	
maternal relationship satisfaction	-2.7	1.03	− 0.25**	
partner relationship satisfaction	− 0.19	0.63	− 0.03	
partner stress	2.2	0.69	0.34**	
maternal stress	− 0.44	0.74	− 0.06	
maternal depression	0.04	0.10	0.04	
maternal anxiety	0.42	1.03	0.04	

*Partner depressive symptoms.* When evaluating continuous EPDS score outcomes for partners, both partner anxiety and partner depression history were significant predictors in step 1. The addition of stress, relationship, and maternal mood variables in step 2 significantly improved the model (r^2^*Δ* = 0.12, p = .005). Maternal relationship satisfaction (β = − 0.25, p = .01) and partner stress (β = 0.34, p = .002) were significant predictors in step 2 (Table 4).

## Discussion

The current study aimed to explore relationship satisfaction, stress, and co-parent mood as predictors of anxiety and depressive symptoms in first-time mothers and their partners in the early postpartum period. Mother-partner dyads were evaluated at two days postpartum. As expected, stress was a predictor of anxiety and depression symptoms in both mothers and partners. Additionally, co-parent anxiety significantly predicted anxiety in both mothers and partners. With regard to social support measures, only maternal perceptions of support were significant predictors, with maternal relationship satisfaction was a predictor of the partner’s depressive symptoms, and maternal perceptions of partner infant support predicted maternal depressive symptoms. Together, these results suggest that stress, relationship satisfaction, and co-parent mood are related, though inconsistently, to depressive and anxious symptoms in mothers and partners.

The findings of this study add to the growing literature evaluating predictors of partner/father mental health in the postpartum period. The findings converge with previous work in partner/father mental health on the role of depression history and stress as predictors of depressive symptoms [9–13]. The findings in this study connecting maternal relationship satisfaction to partner depression, and maternal anxiety to partner anxiety, suggest a need to continue to include maternal and relational variables in future studies exploring paternal/partner depression and anxiety.

Importantly, the findings of this study also suggest a differential role of relationship satisfaction for mothers’ and fathers’ depressive symptoms. Insufficient partner support has been found to increase the risk of co-parent postpartum depression [16, 17], with similar findings in studies of relationship satisfaction predicting maternal depression [29] and paternal depression [11, 30] studied separately. A recently published work with a large sample of parent couples in China found that fathers’ empathy was a significant moderator of the relationships among mothers’ parenting stress, depression, and marital relationship quality [31]. Fewer studies have evaluated relationship satisfaction and anxiety, but longitudinal evidence from one study suggests a predictive role for marital satisfaction in postpartum anxiety symptoms [7]. The differential role of relationship satisfaction and instrumental infant care support found in the current study provides interesting insights and future research directions. These findings suggest that different domains of support and satisfaction are important to measure in both mothers and partners, as they may be of different value for each.

The relationship found between maternal and partner anxiety, but not depression, is partly consistent with previous findings exploring dyadic mood and anxiety symptoms. Mixed results have been found when exploring the relationship between maternal and partner depressive symptoms. On one hand, early postpartum surveys evaluating depressive symptoms found that stress and partner’s depressive symptoms were predictive of both mother and father depressive symptoms [9]; on the other hand, longitudinal surveys of partners found fathers’ prenatal depression predicted worsening maternal depression, but the opposite relationship was not found for paternal depression [32]. Complicating the relationship even further, a recent longitudinal cohort study of couples found no relationship between maternal and paternal depression, but only found relationships among social support and depression, anxiety, and stress for both parents [33]. A recent meta-analysis estimated concurrent perinatal depression in mothers and fathers at up to 3.18% [34]. Importantly in the current study, it was not partners’ relationship satisfaction, but the mother’s satisfaction, that predicted partner depressive symptoms. Being able to differentiate the individual’s satisfaction from the co-parent’s rated satisfaction is an important feature of dyadic studies, and a reason to continue exploring mother and partner mental health in a couple framework.

The findings of this study should be considered in light of the following limitations. First, at a single timepoint, evidence for the directionality of the relationships cannot be determined. Maternal dissatisfaction with infant care, for example, could be influenced by depressive symptoms leading to more negative perceptions of support, or symptoms leading to greater support needs and expectations than mothers with fewer depressive symptoms. Future research using a longitudinal approach to these questions would help to complement and explicate the relationships found in this study. Additionally, the early postpartum period is a time known for emotional lability, and depressive and anxious symptoms assessed at this time should be viewed differently than the same symptoms seen prenatally or at later postpartum weeks. Recent research suggests that the EPDS at early postpartum is not reliably able to differentiate between diagnosable postpartum depression and adjustment disorder, but it is a useful tool in differentiating both trajectories from healthy controls [35]. Importantly, evidence suggests that EPDS scores within the first two days to one week postpartum have a strong predictive power for detecting later postpartum depression [36, 37]. Taken together, these findings suggest that depressive and anxious symptoms at this early timepoint require careful assessment and follow-up to determine if they will continue and develop into anxiety or depressive disorders. Finally, the available sample at the partner hospital was comprised of primarily white, affluent, heterosexual couples, limiting the ability to generalize these findings broadly to other couples and to single parents.

Future research is needed to continue to understand two key areas of postpartum mental health: the risk factors and potential preventive strategies that improve paternal or partner mental health, and the potential impact of mental health interventions or preventive programs that treat the couple as a dyad, rather than an isolated focus on the mother.

These findings add to a growing body of literature on mother-partner mental health that have important implications for practice. Live-in partners are uniquely positioned to be influential in detecting, facilitating treatment, and supporting postpartum depression and anxiety, yet partners report feeling unequipped for this role [38] and are not commonly included in interventions designed to prevent or treat postpartum mood concerns. Further, increasing evidence is pointing to the unmet need of detection and treatment of partner/paternal depression and anxiety in the postpartum period [8]. The relationship found between mothers’ and partners’ anxiety symptoms in this study underscore the importance of considering both parents’ mental health when developing optimal follow up care in the postpartum period. Considering models of care that directly incorporate partners in support, prevention, detection, and treatment of mood disorders in the postpartum period may produce innovative and effective programs for co-parent couples.

## Data Availability

Current study deidentified data was collected as a pre-randomization baseline assessment for an ongoing clinical trial of a breastfeeding intervention, available at clinicaltrials.gov #NCT04578925.

## References

[1] Gavin NI, Gaynes BN, Lohr KN, Meltzer-Brody S, Gartlehner G, Swinson T (2005). Perinatal depression: a systematic review of prevalence and incidence. Obstet Gynecol.

[2] Vesga-López O, Blanco C, Keyes K, Olfson M, Grant BF, Hasin DS (2008). Psychiatric Disord in pregnant and postpartum women in the United States. Arch Gen Psychiatry.

[3] Rogers A, Obst S, Teague SJ, Rossen L, Spry EA, Macdonald JA, Sunderland M, Olsson CA, Youssef G, Hutchinson D (2020). Association between maternal perinatal depression and anxiety and child and adolescent development: a meta-analysis. JAMA Pediatr.

[4] Field T (2017). Prenatal depression risk factors, developmental effects and interventions: a review. J Pregnancy Child Health.

[5] Lancaster CA, Gold KJ, Flynn HA, Yoo H, Marcus SM, Davis MM (2010). Risk factors for depressive symptoms during pregnancy: a systematic review. Am J Obstet Gynecol.

[6] Biaggi A, Conroy S, Pawlby S, Pariante CM (2016). Identifying the women at risk of antenatal anxiety and depression: a systematic review. J Affect Disord.

[7] Clout D, Brown R (2016). Marital relationship and attachment predictors of postpartum stress, anxiety, and depression symptoms. J Soci Clin Psychol.

[8] Rao WW, Zhu XM, Zong QQ, Zhang Q, Hall BJ, Ungvari GS, Xiang YT (2020). Prevalence of prenatal and postpartum depression in fathers: a comprehensive meta-analysis of observational surveys. J Affect Disord.

[9] Anding JE, Röhrle B, Grieshop M, Schücking B, Christiansen H (2016). Couple comorbidity and correlates of postnatal depressive symptoms in mothers and fathers in the first two weeks following delivery. J Affect Disord.

[10] Bergström M (2013). Depressive symptoms in new first-time fathers: associations with age, sociodemographic characteristics, and antenatal psychological well-being. Birth.

[11] Gawlik S, Muller M, Hoffmann L, Dienes A, Wallwiener M, Sohn C, Schlehe B, Reck C (2014). Prevalence of paternal perinatal depressiveness and its link to partnership satisfaction and birth concerns. Arch Womens Ment Health.

[12] Howarth AM, Swain NR (2020). Predictors of postpartum depression in first-time fathers. Australas Psychiatry.

[13] Philpott LF, Leahy-Warren P, FitzGerald S, Savage E. Prevalence and associated factors of paternal stress, anxiety, and depression symptoms in the early postnatal period.Glob Ment Health. 2022;1–16. 10.1017/gmh.2022.33 PMC9768414 36561920

[14] Barooj-Kiakalaee O, Hosseini SH, Mohammadpour-Tahmtan RA, Hosseini-Tabaghdehi M, Jahanfar S, Esmaeili-Douki Z, Shahhosseini Z (2022). Paternal postpartum depression’s relationship to maternal pre and postpartum depression, and father-mother dyads marital satisfaction: a structural equation model analysis of a longitudinal study. J Affect Disord.

[15] Freitas CJ, Williams-Reade J, Distelberg B, Fox CA, Lister Z (2016). Paternal depression during pregnancy and postpartum: an international Delphi study. J Affect Disord.

[16] Nakamura A, Sutter-Dallay A-L, El-Khoury Lesueur F (2020). Informal and formal social support during pregnancy and joint maternal and paternal postnatal depression: data from the french representative ELFE cohort study. Int J Soc Psychiatry.

[17] Ngai FW, Ngu SF (2015). Predictors of maternal and paternal depressive symptoms at postpartum. J Psychos Res.

[18] Muñoz RF, McQuaid JR, González GM, Dimas J, Rosales VA (1998). Depression screening in a women’s clinic: using automated spanish- and English-language voice recognition. J Consult Clin Psychol.

[19] Cox JL, Holden JM, Sagovsky R (1987). Detection of postnatal depression: development of the 10-item Edinburgh postnatal depression scale. Br J Psychiatry.

[20] Dennis CL, Ross L (2006). Women’s perceptions of partner support and conflict in the development of postpartum depressive symptoms. J Adv Nurs.

[21] O’Connor E, Rossom RC, Henninger M, Groom HC, Burda BU. Primary care screening for and treatment of depression in pregnant and postpartum women: Evidence report and systematic review for the US Preventive Services Task Force. JAMA. 2016 Jan 26;315(4):388–406. doi: 10.1001/jama.2015.18948. PMID: 26813212. 10.1001/jama.2015.18948 26813212

[22] Zelkowitz P, Milet TH (1996). Postpartum psychiatric Disord: their relationship to psychological adjustment and marital satisfaction in the spouses. J Abnorm Psychol.

[23] Edmondson OJ, Psychogiou L, Vlachos H, Netsi E, Ramchandani PG (2010). Depression in fathers in the postnatal period: assessment of the Edinburgh postnatal depression scale as a screening measure. J Affect Disord.

[24] Matthey S, Barnett B, Kavanagh DJ, Howie P (2001). Validation of the Edinburgh postnatal depression scale for men, and comparison of item endorsement with their partners. J Affect Disord.

[25] Robins LN, Helzer JE, Croughan J, Ratcliff KS, National Institute of Mental Health Diagnostic Interview Schedule (1981). Its history, characteristics, and validity. Arch Gen Psychiatry.

[26] Vázquez FL, Muñoz RF, Blanco V, López M (2008). Validation of Muñoz’s Mood Screener in a nonclinical spanish population. Eur J Psychol Assess.

[27] Antony MM, Bieling PJ, Cox BJ, Enns MW, Swinson RP (1998). Psychometric properties of the 42-item and 21-item versions of the Depression anxiety stress Scales in clinical groups and a community sample. Psychol Assess.

[28] Davis MH, Morris MM, Kraus LA (1998). Relationship-specific and global perceptions of social support: associations with well-being and attachment. J Pers Soc Psychol.

[29] Serhan N, Ege E, Ayrancı U, Kosgeroglu N (2013). Prevalence of postpartum depression in mothers and fathers and its correlates. J Clin Nurs.

[30] Wang D, Li YL, Qiu D, Xiao SY (2021). Factors influencing paternal postpartum depression: a systematic review and meta-analysis. J Affect Disord.

[31] Dong S, Dong Q, Chen H (2022). Mothers’ parenting stress, depression, marital conflict, and marital satisfaction: the moderating effect of fathers’ empathy tendency. J Affect Disord.

[32] Paulson JF, Bazemore SD, Goodman JH, Leiferman JA (2016). The course and interrelationship of maternal and paternal perinatal depression. Arch Womens Ment Health.

[33] Dixson BJW, Borg D, Rae KM (2022). The social predictors of paternal antenatal mental health and their associations with maternal mental health in the queensland family cohort prospective study. Arch Womens Ment Health.

[34] Smythe KL, Petersen I, Schartau P. Prevalence of perinatal depression and anxiety in both parents: A systematic review and meta-analysis. JAMA Netw Open. 2022 Jun 1;5(6):e2218969. doi: 10.1001/jamanetworkopen.2022.18969. 10.1001/jamanetworkopen.2022.18969 PMC9233234 35749112

[35] Hahn L, Eickhoff SB, Habel U (2021). Early identification of postpartum depression using demographic, clinical, and digital phenotyping. Transl Psychiatry.

[36] El-Hachem C, Rohayem J, Bou Khalil R (2014). Early identification of women at risk of postpartum depression using the Edinburgh postnatal depression scale (EPDS) in a sample of lebanese women. BMC Psychiatry.

[37] Dennis CL (2004). Can we identify mothers at risk for postpartum depression in the immediate postpartum period using the Edinburgh postnatal depression scale?. J Affect Disord.

[38] Henshaw EJ, Durkin KM, Snell RJ (2016). First-time parents’ shared representation of postpartum depressive symptoms: a qualitative analysis. Soc Sci Med.

